# Pentraxin 3 (PTX3) Expression in Allergic Asthmatic Airways: Role in Airway Smooth Muscle Migration and Chemokine Production

**DOI:** 10.1371/journal.pone.0034965

**Published:** 2012-04-18

**Authors:** Jingbo Zhang, Lianyu Shan, Latifa Koussih, Naresh Singh Redhu, Andrew J. Halayko, Jamila Chakir, Abdelilah S. Gounni

**Affiliations:** 1 Department of Immunology, Faculty of Medicine, University of Manitoba, Winnipeg, Manitoba, Canada; 2 Department of Physiology, Faculty of Medicine, University of Manitoba, Winnipeg, Manitoba, Canada; 3 Institut Universitaire de Cardiologie et de Pneumologie de Québec, Université Laval, Sainte-Foy, Québec, Canada; 4 Xinqiao Hospital, Third Military Medical University, Chongqing, China; University of Pittsburgh, United States of America

## Abstract

**Background:**

Pentraxin 3 (PTX3) is a soluble pattern recognition receptor with non-redundant functions in inflammation and innate immunity. PTX3 is produced by immune and structural cells. However, very little is known about the expression of PTX3 and its role in allergic asthma.

**Objectives and Methods:**

We sought to determine the PTX3 expression in asthmatic airways and its function in human airway smooth muscle cells (HASMC). *In vivo* PTX3 expression in bronchial biopsies of mild, moderate and severe asthmatics was analyzed by immunohistochemistry. PTX3 mRNA and protein were measured by real-time RT-PCR and ELISA, respectively. Proliferation and migration were examined using ^3^H-thymidine incorporation, cell count and Boyden chamber assays.

**Results:**

PTX3 immunoreactivity was increased in bronchial tissues of allergic asthmatics compared to healthy controls, and mainly localized in the smooth muscle bundle. PTX3 protein was expressed constitutively by HASMC and was significantly up-regulated by TNF, and IL-1β but not by Th2 (IL-4, IL-9, IL-13), Th1 (IFN-γ), or Th-17 (IL-17) cytokines. *In vitro*, HASMC released significantly higher levels of PTX3 at the baseline and upon TNF stimulation compared to airway epithelial cells (EC). Moreover, PTX3 induced CCL11/eotaxin-1 release whilst inhibited the fibroblast growth factor-2 (FGF-2)-driven HASMC chemotactic activity.

**Conclusions:**

Our data provide the first evidence that PTX3 expression is increased in asthmatic airways. HASMC can both produce and respond to PTX3. PTX3 is a potent inhibitor of HASMC migration induced by FGF-2 and can upregulate CCL11/eotaxin-1 release. These results raise the possibility that PTX3 may play a dual role in allergic asthma.

## Introduction

Asthma is a chronic inflammatory condition of the airways characterized by bronchial hyperresponsiveness, infiltration of inflammatory cells, and airway remodeling. Increased airway smooth muscle mass and deposition of extracellular matrix (ECM) proteins, is an important feature of airway remodeling [1]. Cumulative evidence suggest that airway smooth muscle cells (ASMC) are not only the target for but also a rich source of different pro-asthmatic factors [2]. These studies indeed suggest that ASMC may be classified as ‘inflammatory-like’ cells by virtue of their capacity to produce and respond to multiple pro-inflammatory mediators [2], [3], [4]. These mediators in turn modulate the ASMC functions such as cell proliferation, migration, and matrix production, eventually leading to airway remodeling. Importantly, it is now evident that ASMC can directly respond to inhaled environmental factors via mechanisms that are either immune-mediated such as both high- and low-affinity IgE receptors [5], [6], [7], [8], [9], or in an immune-independent fashion such as protease activated receptor-2 (PAR2) or calreticulin [10], [11], summarized in [12]. Targeting ASM, therefore, presents an attractive treatment regimen for chronic airway inflammatory diseases.

Pentraxin 3 (PTX3) is a newly discovered member of the long pentraxins, and an acute phase protein that has emerged as a new serological marker reflecting tissue inflammation and damage under diverse human pathological conditions [13], [14], such as acute myocardial infarction [15], small vessel vasculitis [16], rheumatoid arthritis [17], chronic kidney disease [18], septic shock and tuberculosis [19], [20]. PTX3 can be produced by a variety of cells at the site of infection or inflammation. These include macrophages, dendritic cells [21], [22], neutrophils [23], endothelial cells [24], fibroblasts [25], epithelial cells [26], and vascular smooth muscle cells [27]. As a soluble pattern recognition receptor, PTX3 binds to and plays non-redundant protective role against selected pathogens, such as fungi, bacteria and virus [28], [29], [30]. It orchestrates complement activation, and displays opsonic activity by promoting phagocytosis and clearance of the pathogens. PTX3 is also a component of ECM protein required for the assembly of the cumulus oophorus and female fertility [31], thereby linking its function to the ECM remodeling. Moreover, PTX3 interacts with other biologically active molecules, such as fibroblast growth factor-2 (FGF-2) [32] and P-selectin [33].

Considering an ‘inflammatory-like’ phenotype of ASMC [2], we hypothesized that PTX3 may also be involved in airway diseases such as allergic asthma. Thus, we first investigated the expression of PTX3 in bronchial biopsies of asthmatic patients, and then analyzed the production of PTX3 in human primary ASMC and epithelial cells (EC) i*n vitro*. We further investigated the possible effects of recombinant human PTX3 on ASMC functions such as inflammatory mediator release, cell migration, and proliferation.

## Results

### Expression of PTX3 is increased in bronchial biopsies of allergic asthmatic patients

**Figure 1:**
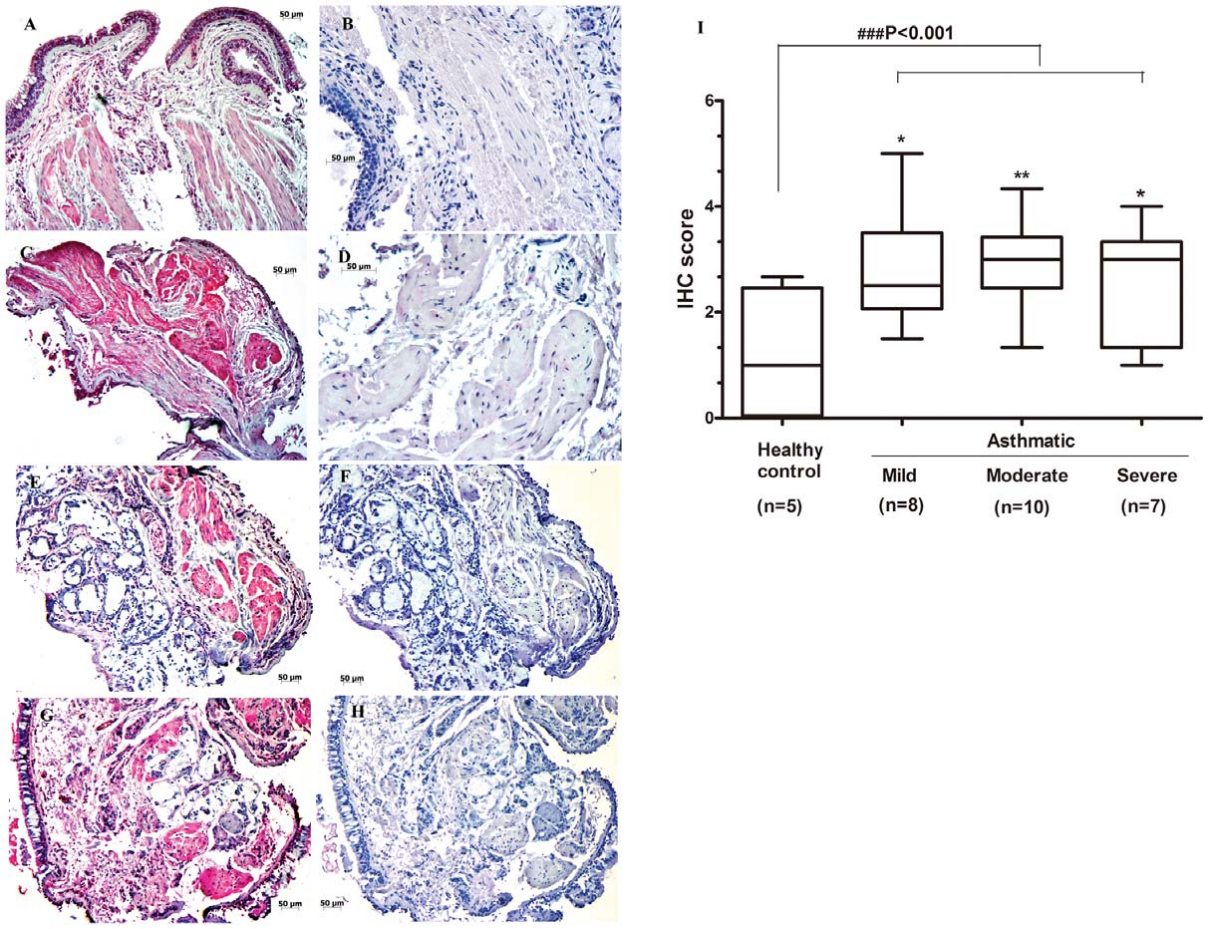
PTX3 is increased in human allergic asthma. Positive PTX3-immunoreactivity is shown from representative bronchial biopsies of healthy control (A), mild (C), moderate (E), and severe (G) asthma subjects. No staining was observed in isotype-matched control mAbs in corresponding biopsies (B, D, F, or H). The scores of PTX3 immunoreactivity is shown (I). ###P<0.001 all allergic asthmatic groups *vs* healthy control; *P<0.05, **P<0.01 each asthmatic subgroup *vs* healthy control. (Original magnifications ×100). Red staining indicates PTX3 expression.

Bronchial biopsy specimens from healthy controls (n = 10) and subjects with allergic asthma (n = 27; 9 with mild, 10 with moderate, and 8 with severe asthma) were used for immunohistochemistry staining using mAb anti-human PTX3. Positive PTX3 immunoreactivity was detected in all cases in the epithelium, bronchial mucosa inflammatory infiltrating cells and ASM cell layers (Figure 1 A, C, E and G). Substitution of the first Ab with matched rat-IgG2b control eliminated the positive signal, demonstrating the specificity of the analysis (Figure 1 B, D, F and H).

Interestingly, since PTX3 was abundantly present at sites of ASM bundle, we performed semi-quantitative assessment through blind grading [34]. PTX3 staining intensity was significantly increased in asthmatic groups compared to healthy controls (Figure 1 I, P<0.001). A significantly higher staining intensity was found in mild (Figure 1 C) (P<0.05), moderate (Figure 1 E) (P<0.01) or severe (Figure 1 G) (P<0.05) asthmatic subjects compared to healthy controls (Figure 1 A). Furthermore, comparing the mean values of PTX3 staining intensity in the subgroups of asthmatic subjects revealed that severe and moderate group had higher intensity than mild asthmatic group without reaching statistical significance (P>0.05).

### PTX3 protein is highly expressed by human ASMC (HASMC) *in vitro*

**Figure 2:**
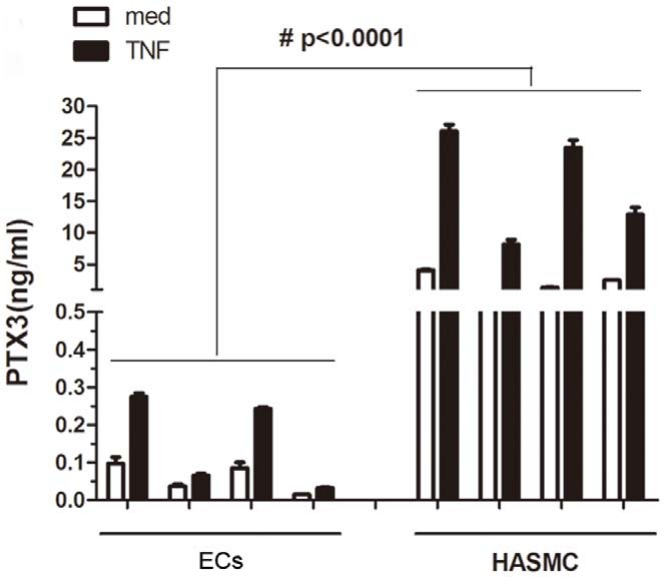
HASMC produce enhanced PTX3 *in vitro* compared to airway EC. (A) 75–80% confluent HASMC or ECs were growth arrested by FBS deprivation for 48 h, and stimulated in fresh FBS-free medium containing TNF (10 ng/ml) or medium alone. Supernatants were collected at 24 h, and PTX3 was quantified using ELISA. #P<0.0001 PTX3 protein release from HASMCs versus from airway ECs by ANOVA.

Lung EC lines have been previously shown to express PTX3 [26]. Similarly, PTX3 immunoreactivity was observed in our study in both ECs and HASMC within bronchial biopsies of asthmatic and healthy donors (Figure 1). We then decided to compare PTX3 release from HASMC (n = 4 donors) with that in human airway ECs (n = 4 donors). Under similar culture conditions and the same cell number, levels of PTX3 released from cultured HASMC was far greater than the airway EC at the baseline (mean value more than 36-fold) or upon TNF stimulation (mean value more than 115-fold) (Figure 2), suggesting that HASMC are one of the main producers of PTX3 in the airways.

### The effect of proinflammatory, Th2, and Th1 cytokines on PTX3 release from HASMC

**Figure 3:**
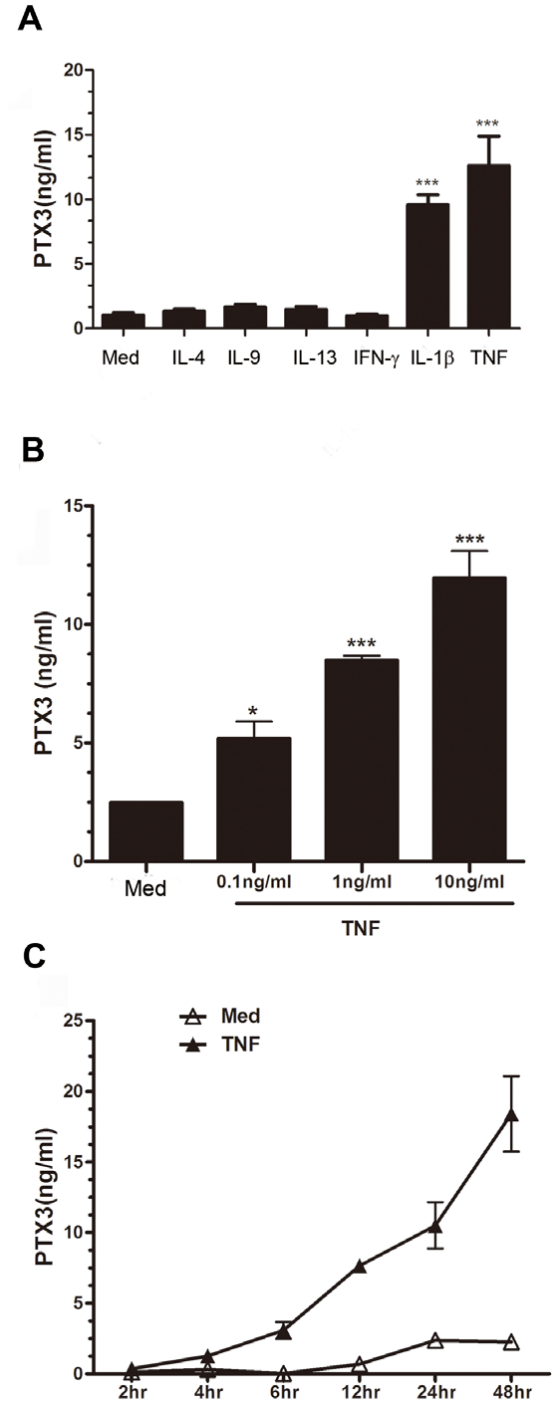
Cytokines induce PTX3 expression in HASMC *in vitro*. (A) PTX3 protein release from HASMC stimulated with proinflammatory (IL-1β, TNF, 10 ng/ml), Th2 (IL-4, IL-9, 10 ng/ml, or IL-13, 100 ng/ml), or Th1 cytokines (IFN-γ, 100 U/ml) for 24 h (n = 3). (B) PTX3 protein release was measured in response to increasing concentrations of TNF at 24 h (n = 5), or (C) at various time-points in presence of TNF (10 ng/ml) (n = 3). *p<0.05, ***p<0.001 compared to medium group.

Considering an inflammatory milieu in allergic asthma [35], we then investigated the effect of proinflammatory, Th2, and Th1 cytokines on PTX3 protein release by HASMC. TNF and IL-1β induced a significant increase in PTX3 protein release from HASMC. However, HASMC stimulated with Th2 (IL-4, IL-9 and IL-13), Th1 (IFN-γ), or Th17 (IL-17) cytokines showed no significant effect on PTX3 release (Figure 3 A, and data not shown).

The central role of TNF in lung inflammation is not only supported by animal models, but also human studies [36], [37]. So, we further characterized the dose or time course effect of TNF on PTX3 expression by HASMC. A statistically significant increase in PTX3 protein release from HASMC occurred with 0.1 ng/ml (*P<0.05), 1 ng/ml (***P<0.001), and 10 ng/ml (***P<0.001) concentration of TNF (n = 5) (Figure 3 B). Furthermore, time course assay showed that PTX3 protein release by HASMC was time-dependent and reached a maximum at 48 h in response to TNF (10 ng/ml) stimulation (Figure 3 C).

### Recombinant PTX3 induces CCL11/eotaxin-1 but not TGFβ1, CXCL8/IL-8, or IL-6 protein release from HASMC

**Figure 4:**
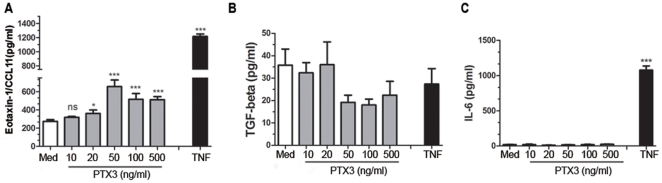
Recombinant human PTX3 induce cytokine release from HASMC. Eotaxin-1/CCL11 (A), TGF-β1 (B), and IL-6 (C) proteins were measured by ELISA upon 24 h stimulation of serum deprived HASMCs with graded concentrations of recombinant PTX3 (*P<0.05, **P<0.01 and ***P<0.001 compared with medium group).

Because the functions of PTX3 are not yet completely defined, we explored whether PTX3 could be an inducer of inflammatory mediators. To test this hypothesis, serum-deprived HASMC were stimulated with a graded concentration of human recombinant PTX3 (10–500 ng/ml). A statistically significant increase in eotaxin-1/CCL11 release from HASMC occurred with 50, 100, and 500 ng/ml PTX3 at 24 h (P<0.05, Figure 4 A). However, PTX3-stimulated HASMC showed no statistical significance in TGFβ1 (Figure 4 B), IL-6 (Figure 4 C) or CXCL8/IL-8 release (p>0.05, n = 3; data not shown). Taken together, the above data demonstrate that PTX3 can induce CCL11/eotaxin-1 production in HASMC but fails to affect TGF-β1, IL-6 or CXCL8/IL-8.

### Recombinant PTX3 inhibits FGF2-induced chemotaxis but not proliferation in HASMC

**Figure 5:**
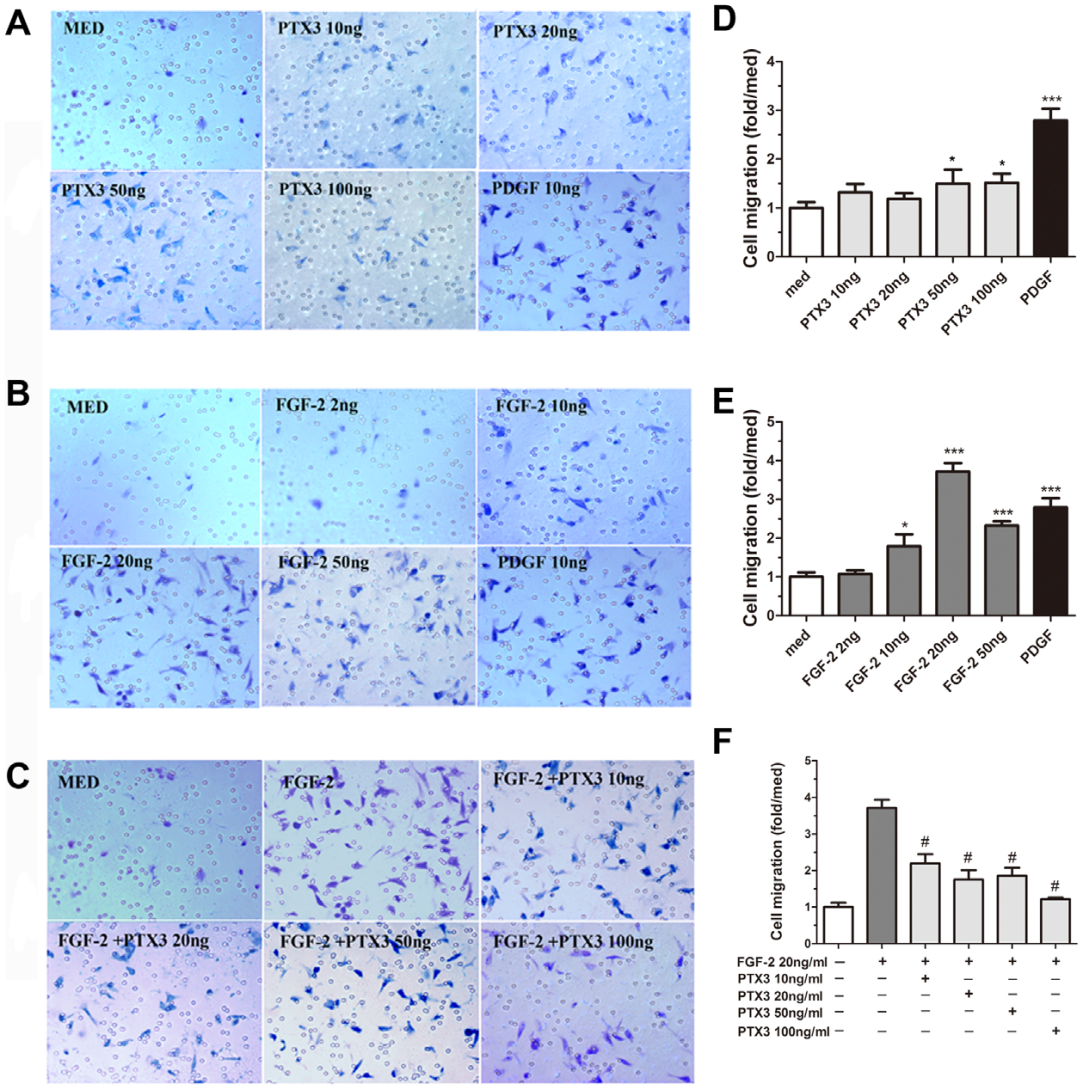
PTX3 abrogates FGF-2 induced HASMC migration. Boyden upper chamber-seeded HASMC were exposed to graded concentrations of (A) PTX3, (B) FGF-2, and (C) PTX3 plus FGF-2 (20 ng/ml) in lower compartment for 4 h. The migrated cells were quantified by light microscope (magnification, ×200). Migration is expressed as fold increase relative to medium control (D, E, and F). *P<0.05, ***P<0.001 *vs* medium control. #P<0.001 compared to FGF-2 (20 ng/ml). n = 3.

PTX3 was previously shown to play an important role in tissue remodeling and matrix assembly [38]. We therefore investigated its role in HASMC migration as a surrogate marker of airway tissue remodeling. Migration of HASMC was slightly increased by PTX3 alone (50 ng/ml, 1.50±0.40- fold and 100 ng/ml, 1.51±0.32-fold, P<0.05). Platelet-derived growth factor (PDGF, 10 ng/ml), used as positive control, significantly increased the HASMC chemotaxis (3.02±0.65-fold, P<0.001) compared to the medium control (Figure 5 A).

Previous observations have shown the ability of PTX3 to bind FGF-2, thus acting as an FGF-2 antagonist [32], [39]. Furthermore, FGF-2 is known to contribute to HASMC proliferation and migration ability that are believed to be major contributors to airway remodeling in asthma [40]. On this basis, we further investigated the capacity of PTX3 to affect FGF-2-dependent HASMC migration. As showed in. Figure 5 B, FGF-2 significantly increased migration of HASMC, with an optimal response at 20 ng/ml (3.71±0.71-fold, P<0.001 compared to the medium). Concentration of PTX3 between 10–100 ng/ml significantly inhibited the effect of FGF-2 on HASMC migration (Figure 5 C, #P<0.001 compared to FGF-2 alone (20 ng/ml, n = 3).

We then determined whether PTX3 modulates FGF-2 induced HASMC proliferation. Using [^3^H]-thymidine incorporation assays or Alamar Blue™ assay, we found that the PTX3 was incapable to modulate HASMC proliferation alone in contrast to PDGF stimulated cells, used as positive control (data not shown). Furthermore, FGF-2 stimulated HASMC showed significant proliferation as expected. However, FGF-2-mediated HASMC proliferation was not affected by addition of PTX3 (data not shown).

Taken together, these data demonstrate that PTX3 has the ability to inhibit FGF2-induced migration of HASMC at least *in vitro* suggesting its role as potential protective mechanism against remodelling observed in asthma.

## Discussion

The present study is the first description of PTX3 expression in patients with allergic asthma. A significantly higher expression of PTX3 within the ASMC area was observed in mild, moderate, and severe asthmatics compared to healthy donors. PTX3 is abundantly expressed within ASM bundle and epithelial cell layers as well as inflammatory cell infiltrate. Furthermore, at least *in vitro*, primary HASMC produced higher levels of PTX3 at the baseline and upon TNF stimulation compared to epithelial cells, suggesting that HASMC are one of the major sources of this mediator in the airways. We also showed that PTX3 can be up-regulated by TNF, but not IFNγ, IL-17, IL-4, IL-9, or IL-13 in HASMC. Moreover, we showed that the PTX3 induces CCL11/eotaxin-1 release, but not IL-6, CXCL8/IL-8, or TGF-β and counteracts FGF-2-induced HASMC migration. Taken together, our data suggest a potential dual role of PTX3 in allergic asthma by enhancing airway inflammation via local CC chemokine expression from HASMC; and down regulating the remodeling by counteracting FGF-induced HASMC migration.

PTX3 is pleiotropic molecule that has been shown to have antibody like functions such as opsonizing properties, activating and regulating complement and binding to pathogens [13]. Moreover, a potential role of PTX3 in the inflammatory response is suggested by elevated PTX3 levels in plasma of patients with sepsis [41], acute myocardial infarction [15], or chronic kidney disease [18]. Interestingly, PTX3 knockout mice are susceptible to pulmonary aspergilosis [29], whereas PTX3 transgenic mice display greater resistance to cecal ligation and puncture [42]. The human *PTX3* gene is located on the chromosome 3q24–28 [43], the region which has been linked to the phenotype related to Der P1 allergen-specific IgE, and total serum IgE in humans [44]. Therefore, an association between PTX3 levels and allergic airway disease may not be denied. The current study adds to this notion by clearly showing that allergic asthmatic patients express higher PTX3 levels compared to healthy controls. In COPD, an obstructive inflammatory disease with some features similar to asthma, PTX3 expression was found to be reduced in lung sections of patients which correlated with FEV1 (a marker for small airway obstruction); while the PTX3 expression in induced sputum in COPD patients did not have any correlation with FEV1 [45]. Another study showed an enhanced PTX3 expression in induced sputum of COPD patients compared to controls [46]; whereas the PTX3 knockout mice showed no difference in cigarette smoke-induced pulmonary inflammation, emphysema, and body weight changes compared to the wild-type mice [47]. Therefore, a clear role of PTX3 in the pathogenesis of COPD remains yet to be established.

The reason why PTX3 is highly expressed within the airway of asthmatics remains unknown, but there are at least two possible explanations. First, emerging evidence suggests PTX3 as an important structural component of ECM that plays a crucial role in sustaining its stability [13], [14], [48]. In particular, PTX3 is involved in the organization of ECM by cross-linking hyaluronan via interaction with TNF-stimulated gene 6 (TSG6) and Inter-α-trypsin inhibitor (IαI) [49], [50]. In agreement with the role of PTX3 in matrix organization, our IHC staining showed a significant PTX3 staining within the ASM bundles of asthmatic bronchial tissue compared to healthy donors, which may provide another possible explanation of airway remodeling observed in asthma. Second, PTX3 may potentially contribute to airway inflammation and subsequent remodeling. Furthermore, PTX3 enhanced CCL11/eotaxin-1 release in HASMC, chemokine that plays an important role in eosinophil airway inflammation [51], and remodeling [52].

However, it is worth mentioning that CCL11/eotaxin-1, besides its role in attracting eosinophils, can exert a specific regulatory function of neutrophil recruitment *in vivo* as has been shown in a mouse model of endotoxemia [53] via probable down-regulation of CXC chemokine CXCL8/IL-8 [54]. In light of prior evidence and present data, it is tempting to speculate that PTX3-mediated CCL11/eotaxin-1 release may down-regulate exaggerated neutrophilic inflammation by suppressing CXC chemokine release during acute inflammation, hence creating a negative feedback to establish tissue homeostasis especially in severe asthma that is characterized by more neutrophilic infiltrates [55]. In agreement with this possibility, Mantovani and colleagues reported recently that PTX3 dampens neutrophil recruitment *in vivo* via a P-selectin dependent mechanism [33]; and previous data demonstrated that PTX3 knockout mice develop more myocardial damage associated with more neutrophil infiltration in a model of cardiac ischemia reperfusion injury [56]. These data suggest a potential role of PTX3 in down regulating neutrophilic airway inflammation in allergic asthma. Taken together, it is plausible that on one hand a reduced PTX3 expression in COPD favors a characteristic neutrophilic response [45], whereas enhanced PTX3 expression in asthmatics may embark on an eosinophilic inflammation potentially via inducing eotaxin-1/CCL11 expression in airway structural cells. More studies are needed further to investigate these possibilities.

We also found that PTX3 significantly inhibits FGF-2-induced chemotaxis in HASMC. Furthermore, in contrast with previous study in vascular smooth muscle cells [39], we did not find any effect of PTX3 protein on HASMC proliferation, suggesting that PTX3 effect is cell specific. Although PTX3 at higher doses by itself tend to induce ASM migration, it attenuates the FGF-2-induced migration. It may be plausible that the locally-produced PTX3 facilitates immune inflammatory cell recruitment, while counteracts the effects of FGF-2 in inducing migration of ASM itself. Moreover, since PTX3 has been shown to act as an antagonist of FGF-2 by others [32], [39], it may be plausible that the inhibitory effect on migration is selective and specific to FGF-2-induced migration. We are exploring these hypotheses further by extensive *in vitro* studies and by employing PTX3 knockout mouse model. Besides EC that is the main source of FGF-2 in asthmatic airways, inflammatory cells mobilized into the airways such as eosinophils, macrophages, T-cells, mast cells are also potent producers of FGF-2 [40]. FGF-2 is known to be released directly following allergen-induced degranulation of mast cells which may cause HASMC migration [57]. On this basis, it is enticing to speculate that PTX3 released from HASMC, as well as others sources, may have a regulatory role in smooth muscle migration and thus airway remodeling.

In conclusion, this novel study demonstrated an increased PTX3 expression in bronchial biopsies of allergic asthmatics compared to healthy controls. Considering HASMC as one of the major cell types in the airway, constitutive expression and inducible PTX3 produced from this source may play a dual role in inflammatory and airway remodelling process in allergic asthma.

Some of the results of these studies have been previously presented in American Thoracic Society International Conference and reported in the form of an abstract online.

## Materials and Methods

### Ethics statement

Human airway smooth muscle cells (HASMC) were obtained from surgical patients following the receipt of written informed consent in accordance with procedures approved by the Human Research Ethics Board of the University of Manitoba, Winnipeg, Canada. Bronchoscopy to obtain lung tissue (for immunohistochemistry) was performed in accordance with procedures approved by the Human Research Ethics Board of Laval University, Quebec, Canada, following the receipt of the subjects' written informed consent. The current study was approved by the research ethics boards of the University of Manitoba, Winnipeg, and the Laval University, Quebec, Canada.

### Reagents

Recombinant human TNF, IL-1β, IL-4, IL-9, IL-13, IFN-γ, FGF-2, PTX3 proteins, and ELISA kits for human PTX3 were purchased from R&D Systems (Minneapolis, MN). Monoclonal rat anti-PTX3 (MNB1) was purchased from ALEXIS Biochemicals (Lausen, Switzerland). All other reagents were procured from Sigma-Aldrich Canada Ltd. (Oakville, ON), unless specified.

### Subjects

Asthmatic subjects were fulfilling the American Thoracic Society selection criteria [58]. Mild asthmatics had an FEV1 of >80% predicted, used no inhaled corticosteroids (CS), had symptoms <2 days per week, and rare exacerbations. Moderate asthmatics used inhaled CS on a daily basis and had an FEV1 of >50% predicted and used less than 1000 µg fluticasone/day. Furthermore, moderate asthmatics have a history of emergency department (ED)/urgent care visits or systemic steroid use within the past year. Severe asthmatics needed treatment with high dose of inhaled CS (>1000 µg fluticasone/day) or systemic CS. All asthmatic subjects were atopic with at least one positive response to common allergens on allergy skin prick tests. In the month preceding the study, none of the subjects reported a respiratory infection or an increase in asthma symptoms. Some subjects who had the smoking history had quit at least three months. Healthy subjects were non-atopic non-smokers with no history of asthma, respiratory disease or atopy, except one who had a smoking history and had quit 2.5 years ago. All healthy subjects showed normal pulmonary function testing and a negative methacholine challenge. Subjects' characteristics are shown in Table 1.

**Table 1:** Clinical characteristics of the subjects.

Category	Subcategory	Healthy controls	Mild asthma	Moderate asthma	Severe asthma
Number (n)		10	9	10	8
†Age (years)		24±1.5	24±1.6	33±4.5	39±5.9
Sex	Male	7	4	2	1
	Female	3	5	8	7
Smoking status	NS	8	6	9	5
	Ex	2	3	1	3
	S	0	0	0	0
Atopy	Yes	0	9	10	8
	No	10	0	0	0
†FEV1 (%)		98.5±2.8	96.0±4.4	86.3±6.7	78.9±6.0
†PC_20_ (mg/ml)		111.4±18	1.7±0.7	0.7±0.5	-
Asthma medication	ICS (µg)	-	-	437.5±100.2	1187.5±277.2
	LABA	-	-	-	Yes

† Data are expressed as mean ± SEM.

NS: Non smoker; Ex: Ex-smoker; S: Smoker.

FEV_1_: forced expiratory volume in 1 second.

LABA: Long acting β_2_-agonists.

### Immunohistochemistry

Paraffin sections were stained with rat anti-human PTX3 mAb or control IgG2b followed by secondary biotin goat anti-rat IgG and streptavidin-alkaline phosphatase developed by fast red as described earlier [59]. Briefly, formalin-fixed tissues were paraffin embedded, and 5-µm-thick sections were prepared, deparaffinized in xylene, and rehydrated through graded concentrations of alcohol to water and then boiled with microwave for 10 min in sodium citrate buffer (pH, 6.0) for antigen retrieval. Sections were washed and then incubated with blocking solution (5% goat serum, 1% BSA, 0.1% cold fish skin gelatin in PBS) for 60 min at room temperature. Rat anti-human PTX3 mAb or control mouse IgG2b (both at 10 µg/ml) were added, and sections were incubated overnight at 4°C. Slides were then washed twice with PBS followed by incubation for 1 h at room temperature with biotin-conjugated goat anti-rat IgG (Jackson ImmunoResearch Laboratories, Inc. West Grove, PA). Slides were then washed extensively with PBS and incubated with streptavidin-alkaline phosphatase for 30 min at room temperature. After washing with PBS, the slides were developed using Fast Red and counterstained with Mayer's hematoxylin. Positive cells stained red after development with Fast Red (Sigma-Aldrich Canada Ltd., Oakville, ON). Isotype-matched control mAb was used for negative control. Semi-quantitative assessment of specimens were performed by three independent observers and a pathologist in a blinded manner as previously described [34]: 0, absent or faint staining of an occasional ASM bundle only; l+, faint staining of several ASM bundle; 2+, moderate intensity staining of most ASM bundle; 3+, intense staining of most ASM bundle and 4+ intense staining of all ASM bundle.

### HASMC preparation

HASMC were obtained as described previously [59], [60], [61] from macroscopically healthy segments of the trachea after lung resection from surgical patients in accordance with procedures approved by the Human Research Ethics Board of the University of Manitoba, Winnipeg, Canada. Primary HASMC were isolated from explants. HASMC retain smooth muscle-specific actin, SM22, calponin protein expression, and mobilize intracellular Ca^2+^ in response to acetylcholine [60], and were used at passages 3–5. HASMC were grown on uncoated plastic dishes in complete DMEM (DMEM supplemented with 100 µg/ml streptomycin, 100 U/ml penicillin, and 10% fetal bovine serum). Unless otherwise mentioned, cells were grown to a subconfluent (∼70%) condition and serum starved to synchronize for 48 h in Ham's F12 supplemented with 100 µg/ml streptomycin, 100 U/ml penicillin, and 1X ITS (5 µg/ml insulin, 5 µg/ml transferrin, and 5 ng/ml selenium) before each experiment. Primary bronchial epithelial cells (EC) were isolated from biopsies obtained from healthy controls by using previously described techniques [62], [63]. Briefly, epithelial cells were isolated from bronchial biopsies obtained by bronchoscopy, and were characterized by immunofluorescence and flow cytometry using anti-cytokeratin antibody from Calbiochem (San Diego, CA). This identification confirmed the purity of the bronchial cell culture as we previously described [62], [63]. EC were cultured in DMEM supplemented with 100 µg/ml streptomycin, 100 U/ml penicillin, and 10% fetal bovine serum.

### ELISA

PTX3 ELISA was performed according to the protocol provided by the manufacturer (R&D Systems, Minneapolis, MN). Sensitivity was 20 pg/ml for PTX3. IL-6, IL-8, TGFβ, and eotaxin-1/CCL11 ELISA was performed in-house using matched Abs from R&D Systems. Sensitivity was IL-6 (7.6 pg/ml), CXCL8/IL-8 (7.6 pg/ml), TGF-β (15 pg/ml), and CCL11/eotaxin-1 (10 pg/ml).

### Proliferation and chemotaxis assay

Cell proliferation was examined by using ^3^H-thymidine incorporation [64], cell counting, and Alamar blue assays. Cell migration was analyzed according to the previously described using Boyden chamber assay [65]. Briefly, 48 h serum-starved cells were detached from the culture plate using trypsin (0.5 mg/ml)-EDTA (0.2 mg/ml) solution (Invitrogen Canada Inc., Burlington, ON) and resuspended in Ham's F12 medium containing 100 µg/ml streptomycin, 100 U/ml penicillin, and 1X ITS. A polycarbonate membrane of 8 µm pore size (Neuroprobe, Gaithersburg, MD, USA) was coated with 0.01% collagen type-I in 0.01N HCl solution (Sigma). A 50 µl aliquot of HASMC (5×10^4^ cells/ml) was added to the upper chamber of modified Boyden chamber apparatus (Neuroprobe). In the lower chamber, FGF-2, PDGF, or PTX3 were added as a chemoattractant to the same media as the upper chamber. After 4 h of incubation at 37°C in humidified 5% CO_2_ incubator, the membranes were peeled-off. Cells on the upper side of the membrane were scraped off and the cells migrated to the lower side were fixed and stained with Hemacolor® stain set (EMD Millipore, Billerica, MA, USA) The number of migrated cells was counted in four-five random fields under ×20 magnification by phase contrast microscope (Carl Zeiss Canada Ltd., Toronto, ON).

### Statistical analysis

Data obtained from experiments performed in triplicate and repeated at least three times was represented as means ± SEM. Differences among groups were analyzed using unpaired t-tests or ANOVA together with a post-hoc Bonferroni analysis. Non-parametric data were analyzed using the Kruskal-Wallis test followed by the Mann-Whitney U-test. P values were considered significant at 0.05 levels.
